# Competition among native and invasive *Phragmites australis* populations: An experimental test of the effects of invasion status, genome size, and ploidy level

**DOI:** 10.1002/ece3.5907

**Published:** 2020-01-13

**Authors:** Petr Pyšek, Jan Čuda, Petr Šmilauer, Hana Skálová, Zuzana Chumová, Carla Lambertini, Magdalena Lučanová, Hana Ryšavá, Pavel Trávníček, Kristýna Šemberová, Laura A. Meyerson

**Affiliations:** ^1^ Department of Invasion Ecology Institute of Botany Czech Academy of Sciences Průhonice Czech Republic; ^2^ Department of Ecology Faculty of Science Charles University Prague Czech Republic; ^3^ Department of Ecosystem Biology Faculty of Science University of South Bohemia České Budějovice Czech Republic; ^4^ Department of Evolutionary Biology of Plants Institute of Botany Czech Academy of Sciences Průhonice Czech Republic; ^5^ Department of Botany Faculty of Science Charles University Prague Czech Republic; ^6^ Department of Agricultural and Food Sciences University of Bologna Bologna Italy; ^7^ Department of Botany Faculty of Science University of South Bohemia České Budějovice Czech Republic; ^8^ Department of Natural Resources Science The University of Rhode Island Kingston RI USA

**Keywords:** common reed, Europe, genome size, intraspecific competition, native populations, North America, plant invasion, ploidy level

## Abstract

Among the traits whose relevance for plant invasions has recently been suggested are genome size (the amount of nuclear DNA) and ploidy level. So far, research on the role of genome size in invasiveness has been mostly based on indirect evidence by comparing species with different genome sizes, but how karyological traits influence competition at the intraspecific level remains unknown. We addressed these questions in a common‐garden experiment evaluating the outcome of direct intraspecific competition among 20 populations of *Phragmites australis*, represented by clones collected in North America and Europe, and differing in their status (native and invasive), genome size (small and large), and ploidy levels (tetraploid, hexaploid, or octoploid). Each clone was planted in competition with one of the others in all possible combinations with three replicates in 45‐L pots. Upon harvest, the identity of 21 shoots sampled per pot was revealed by flow cytometry and DNA analysis. Differences in performance were examined using relative proportions of shoots of each clone, ratios of their aboveground biomass, and relative yield total (RYT). The performance of the clones in competition primarily depended on the clone status (native vs. invasive). Measured in terms of shoot number or aboveground biomass, the strongest signal observed was that North American native clones always lost in competition to the other two groups. In addition, North American native clones were suppressed by European natives to a similar degree as by North American invasives. North American invasive clones had the largest average shoot biomass, but only by a limited, nonsignificant difference due to genome size. There was no effect of ploidy on competition. Since the North American invaders of European origin are able to outcompete the native North American clones, we suggest that their high competitiveness acts as an important driver in the early stages of their invasion.

## Graphical abstract

We address experimentally, for the first time, competition between three phylogeographic groups of *Phragmites australis*, a globally important species providing ecosystem services in wetlands—we explored competition between native North American populations, invasive populations in North America that were introduced from Europe, and their native European counterparts from which they originated. Based on our previous studies on the role that genome size plays in this particular invasion, we tested the effect of karyological characteristics (genome size and ploidy levels) of competing population on the outcome of competition.

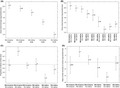

## 1 INTRODUCTION

Research in plant invasions has progressed in recent years, with data accumulated in global databases (Dawson et al., 2017; Pyšek et al., 2017; van Kleunen et al., 2015, 2018) not only allowing improvement of our knowledge about the distribution of naturalized species in world regions, but also facilitating deeper insights into the mechanisms and traits associated with successful invasions (e.g., Guo, van Kleunen, et al., 2018; Razanajatovo et al., 2016). These studies contribute to the existing body of invasion literature focusing on the roles that individual species traits and their interactions have on invasions (van Kleunen, Weber, & Fischer, 2010; Küster, Kühn, Bruelheide, & Klotz, 2008; Pyšek & Richardson, 2007), acting in concert with other factors (Dawson, Burslem, & Hulme, 2011; Pyšek et al., 2015), and depending on the stage of the invasion process (Divíšek et al., 2018; Moodley, Geerts, Richardson, & Wilson, 2013; Pyšek et al., 2009).

The vast majority of such studies search for the determinants of invasiveness by comparing different species, focusing on regional floras (e.g., Pyšek et al., 2012; Inderjit et al., 2018) or taxonomically narrowed study systems (e.g., Gallagher et al., 2011; Grotkopp, Rejmánek, & Rost, 2002). Recently, it is increasingly recognized that addressing invasions at the intraspecific level can provide novel insights into mechanisms underlying plant invasiveness, by comparing particular invading populations and addressing more subtle differences in their performance when trait differences associated with species level are held constant (Cronin, Bhattarai, Allen, & Meyerson, 2015; Pyšek et al., 2019). One system that provides opportunities to focus on organizational levels below the species rank, such as subspecies, populations, or individual genotypes, is a dominant species of wetlands all over the world *Phragmites australis* (common reed, Poaceae; Meyerson & Cronin, 2013; Meyerson, Lambert, & Saltonstall, 2010; Packer, Meyerson, Skálová, Pyšek, & Kueffer, 2017). This grass makes an ideal model system to study invasions by particular populations representing distinct genotypes, with native and invasive populations coexisting within the same geographic range (Eller et al., 2017; Meyerson, Cronin, & Pyšek, 2016; Packer et al., 2017; Pyšek et al., 2018). Although the analogous situation has been described for other tall grass‐like species and grasses, such as in the *Typha* genus (Ciotir & Freeland, 2016) or for *Phalaris arundinacea* (Lavergne, Muenke, & Molofsky, 2010), respectively, and some forbs (e.g., *Myriophyllum spicatum*; Zuellig & Thum, 2012), the common reed invasion in the North America is by far best researched with a great body of accumulated information providing a broad background for ongoing studies (Chambers, Meyerson, & Saltonstal., 1999; Eller et al., 2017; Meyerson, Cronin, & Pyšek, 2016; Packer et al., 2017).

Until recently, the lack of robust data has meant that plant genome size (the amount of nuclear DNA; Greilhuber, Doležel, Lysak, & Bennett, 2005) was among the traits for which plant invasion science does not have a strong research tradition. Its role in invasions was first suggested using individual genera more than 20 years ago (Rejmánek, 1996), later elaborated for *Pinus* (Grotkopp et al., 2002) and *Artemisia* (Garcia et al., 2008) and confirmed by analyses of multispecies datasets. Several papers demonstrated that naturalized or invasive species tend to have smaller genomes than those that have not successfully naturalized or invaded (Kubešová, Moravcová, Suda, Jarošík, & Pyšek, 2010; Pandit, White, & Pocock, 2014; Pyšek et al., 2015) and that small genomes are significantly overrepresented among invasive taxa (Suda, Meyerson, Leitch, & Pyšek, 2015).

Recently, more robust evidence has started to appear in the literature that small genomes promote invasion in plants by interacting with other traits (Meyerson, Cronin, & Pyšek, 2016; Pyšek et al., 2018) and that the role of genome size may differ during different phases of the invasion process, playing the major role in the naturalization stage (Kubešová et al., 2010). The mechanism underlying these analyses is that species with large genomes are less likely to be invasive (Suda et al., 2015). The theoretical basis for this is provided by the “large genome constraint” hypothesis (Knight, Molinari, & Petrov, 2005), proposing that species with small genomes can attain a much wider range of trait states compared to species with large genomes, and many traits associated with large genomes are not compatible with the characteristics of successful invaders (Suda et al., 2015). Moreover, the importance of the association of genome size with invasiveness was supported by a macroecological study that tested its role together with other traits known to promote invasiveness. This analysis also took into account potentially confounding factors in invasions, such as propagule pressure, and genome size turned out to be one of the variables that explained the naturalization success of central European plant species in North America (Pyšek et al., 2015).

In our previous research (Pyšek et al., 2018), an intercontinental comparison of native and invasive populations of common reed (*Phragmites australis*), we revealed a distinct relationship between genome size and invasiveness at the intraspecific level, similar to that reported for *Phalaris arundinacea* (Lavergne et al., 2010, but see Martinez, Baack, Hovick, & Whitney, 2018). For *P. australis*, monoploid genome size (i.e., the amount of DNA in one chromosome set of an organism that, unlike the holoploid genome size, varies independently of ploidy level; Suda et al., 2015) was the only significant variable that clearly separated the North American native plants from those of European origin. This indicates that European populations successfully invaded North America because, relative to native populations, they had a smaller genome, which was associated with plant traits favoring invasiveness (Pyšek et al., 2018). The current study builds on this background and is based on the following premises:

(a) In North America, invasive populations that were introduced from Europe grow in the same habitats as native populations, outcompeting and replacing them (Meyerson, Saltonstall, & Chambers, 2009; Saltonstall, 2002). This implies that a direct competition between the two groups where they co‐occur could be one of the mechanisms behind this particular invasion. The primary assumption that can be made for such a study system is that the invasive populations will be competitively superior to the native populations they replace. This is based on the suggestion that competition is generally considered an important mechanism of plant invasion (e.g., Daehler, 2003; Gioria & Osborne, 2014; Goldstein & Suding, 2014; Vilà, Williamson, & Lonsdale, 2004), together with other factors such as the availability of open niches, propagule pressure, and disturbances. (b) Invasive populations differ from native populations in a number of growth, physiological, and reproductive traits (Pyšek et al., 2019) that can be related to a small genome, an underlying characteristic separating both groups (Pyšek et al., 2018). (c) Based on this, we hypothesized that small genomes constitute a key advantage in a direct competition between invasive and native populations that is manifested through traits associated with genome size (Suda et al., 2015). As genome size interacts with ploidy levels to affect invasion success (which is negatively related to genome size and positively related to ploidy level; Meyerson, Cronin, Bhattarai, et al., 2016; Pandit et al., 2014; te Beest et al., 2012), both characteristics need to be considered when addressing the competitive performance of populations differing in their karyological makeup.

To test the above hypotheses experimentally, we carried out a common‐garden experiment to evaluate the outcome of intraspecific competition among populations of *Phragmites australis* differing in their status (native vs. invasive), genome size (small vs. large), and ploidy levels (tetraploid, hexaploid, or octoploid). We aimed to reveal whether the effects of genome size and ploidy on competitive hierarchies, if there are any, are direct or mediated via plant traits related to karyological features.

## 2 METHODS

### 2.1 Study species

**Figure 1:**
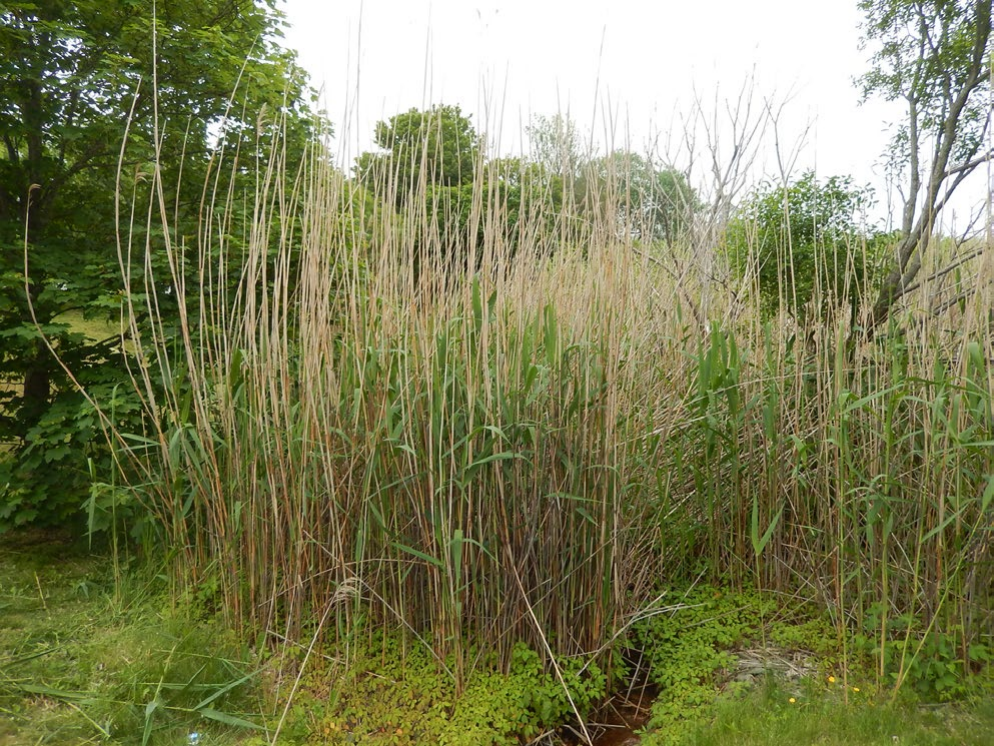
Invasive *Phragmites australis*, Rhode Island, USA. Photo: Petr Pyšek

*Phragmites australis* (Cav.) Trin. ex Steud. (common reed, Poaceae; Figure 1) is tall, helophytic, wind‐pollinated perennial grass with shoots up to 4 m tall, forming an extensive system of rhizomes and stolons (runners), with a single inflorescence developing on each fertile stem, producing 500–2,000 seeds (Packer et al., 2017), but not all shoots are fertile every year and not all seeds fully develop. The species is highly productive (Bittmann, 1953; see Packer et al., 2017, for a review) and exhibits great genetic, karyological, and morphological variation. It belongs to one of the most ploidy‐variable invasive species known, with published cytotypes from 3x to 22x, based on *x* = 12 (te Beest et al., 2012), and there is marked intraspecific variation in genome size (Suda et al., 2015), as well as phylogeographic genetic diversity within the species and the whole genus (Lambertini et al., 2006; Meyerson, Cronin, Bhattarai, et al., 2016; Meyerson et al., 2009; Saltonstall, 2011). *Phragmites australis* colonizes a wide range of environmental conditions (Meyerson, Saltonstall, Windham, Kiviat, & Findlay, 2000) and extends from the tropics to cold temperate regions in both hemispheres, which places it among the world's most cosmopolitan and globally important wild plants providing ecosystem services (Packer et al., 2017). In its confirmed introduced range, which for the European native *P. australis* subsp. *australis* is North America, it is a noxious invader that has converted botanically diverse wetlands into low‐diversity ecosystems where it outcompetes the North American native *P. australis* subsp. *americanus* (Meyerson et al., 2010; Saltonstall, 2002).

### 2.2 Experimental setup

We used *P. australis* clones representing distinct populations, cultivated since 2011 in the Institute of Botany, CAS, in Průhonice, Czech Republic (see Pyšek et al., 2018, 2019, for details on the geographic location from where the plants originated). From April until October, the clones were grown in an experimental garden (49°59′38″ N, 14°33′57″ E), 320 m above sea level in the temperate climate zone, with a mean annual temperature of 8.6°C and precipitation of 610 mm, and for winter transported into the greenhouse to prevent frost damage.

For the experiment, we used 20 clones (see Table 1 and Figure S1) differing in origin (Europe and North America) and status (native and invasive)—the combination of these traits resulted in three groups: native European clones not known to have been introduced to North America (termed “EU‐native”); clones of European origin that were introduced to North America and have become invasive (termed “NA‐invasive”); and North American native clones (“NA‐native”). Further, the clones differed in ploidy levels (4x, 6x, 8x) and, for tetraploids of European origin, both EU‐native and NA‐invasive, also in genome size (small, large). North American native clones were represented only by four tetraploids with large genomes as the small genome was not detected in this group (Pyšek et al., 2018). In NA‐invasive and EU‐native groups, eight clones were used (see Table S1 for the numbers of replicates for particular pairs of competitors). Small genomes varied from 2C = 1.89–1.95 pg, and large genomes varied within 2C = 2.06–2.25 pg (for details on clones used, see Table 1).

**Table 1:** Overview of clones used in the experiment

Clone ID	Origin and status	Ploidy (x)	Genome size (2C‐value in pg)	GS category	Country	Latitude	Longitude	Plants used in the experiment
D615	EU‐native	4	1.89	Small	Russia (Sachalin)	47.03	143.30	55
FRA3	EU‐native	4	1.92	Small	France	44.68	−1.02	56
D620	EU‐native	4	2.06	Large	Spain	40.72	0.58	52
D643	EU‐native	4	2.06	Large	Italy	44.72	11.53	59
D659	EU‐native	6	2.96		Russia (Sachalin)	48.63	142.79	55
D538	EU‐native	6	3.02		Romania	45.00	29.22	56
D589	EU‐native	8	3.88		Romania	45.00	29.22	57
D553	EU‐native	8	3.95		Hungary	47.60	17.03	57
NA134	NA‐invasive	4	1.94	Small	USA (MD)	38.59	−76.05	55
NA94	NA‐invasive	4	1.95	Small	USA (RI)	41.18	−71.57	55
NA96	NA‐invasive	4	2.17	Large	USA (NH)	43.05	−70.90	31
NA159	NA‐invasive	4	2.21	Large	USA (RI)	41.36	−71.64	51
NA224	NA‐invasive	6	3.15		USA (LA)	30.19	−89.54	39
NA148	NA‐invasive	6	3.18		USA (MA)	41.47	−70.76	33
USA2	NA‐invasive	8	3.9		USA (MA)	42.34	−71.09	53
D617	NA‐invasive	8	4.12		USA (RI)	41.79	−71.37	57
NA124	NA‐native	4	2.24	Large	USA (NH)	43.05	−70.90	20
NA61	NA‐native	4	2.25	Large	Canada (NB)	46.07	−64.72	56
NA7	NA‐native	4	2.25	Large	USA (NY)	42.94	−76.74	54
NA8	NA‐native	4	2.3	Large	USA (NY)	42.94	−76.74	45

Note

The clones are arranged by origin and status, ploidy, and genome size. Note that only tetraploids were divided into small and large genome categories, to test the effect of genome size on the outcome of competition.

Nuclear genome size of maternal clones was determined by DNA flow cytometry using Sysmex/Partec CyFlow SL instrument equipped with green (532 nm, 100 mW output power) solid‐state laser. Sample preparation followed the simplified two‐step procedure using Otto buffers as detailed in Doležel et al. (2007). *Bellis perennis* (2C = 3.38 pg; Schönswetter, Suda, Popp, Weiss‐Schneeweiss, & Brochmann, 2007) was chosen as an appropriate internal reference standard. Propidium iodide was used as a stain. Fluorescence intensity of 5,000 particles was recorded during each analysis. Only histograms with coefficient of variation of *G*_0_/*G*_1_ peak of both sample and standard below 3.0% were considered. Each plant was reestimated at least three times on different days. For further details, see Pyšek et al. (2018).

In March 2015, the clones were taken from the collection and transplanted into 90‐L pots filled with sand mixed with 480 g of slow‐release fertilizer Osmocote Pro (release time 12–14 months; ICL Specialty Fertilizers) to propagate the material for the experiment. On 29–30 June 2015, the shoots were cut at about 10 cm above the sand surface, the rhizomes excavated, and ~15‐cm‐long rhizome segments with terminal shoots or buds were cut. The clones were planted in pairs on the opposite sites of 45‐L round pots filled with sand mixed with 240 g of Osmocote Pro. Each clone was planted in competition with one of the others in all possible combinations $\left(\sum_{n=1}^{20}n\right)$ in three replicates (i.e., 630 pots in total). However, one replicate was missing in some combinations with NA‐native clones due to poor growth during multiplication; thus, the experiment was launched with a total of 612 pots. On 20 and 30 July 2015, the plants were checked and those that died were replaced. The plants were watered daily using tap water delivered by an automatic watering system (Hunter Industries). To ensure comparable water supply to all plants, three holes were drilled in each pot 25 cm from the bottom to allow drainage of excessive water and achieve the same water level in each pot. When plants started to exhibit signs of iron deficiency (yellowing), 0.2 g Fe as iron in chelation complex of DTPA dissolved in 150 ml of tap water was added to each pot. All plants were treated with the insecticides Mospilan 20SP and Careo Ultra in the recommended doses at the beginning of the experiment to protect them from unwanted aphid damage. In 2015, plants were grown until full senescence (November), then the aboveground biomass was harvested, the pot surface was covered with spruce brushwood, and the pot sides were wrapped with bubble foil to protect the plants from frost. In early April 2016, the frost protection was removed.

### 2.3 Traits measured

**Figure 2:**
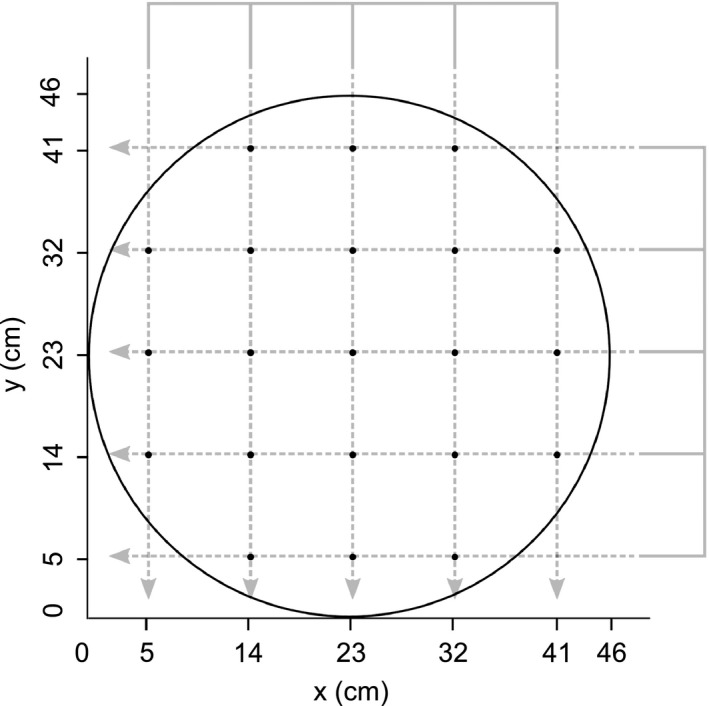
The pattern of shoot selection for harvest. A wire structure was slid into the pot at the substrate surface level from two sides to form a grid that was used to identify the position of harvested shoots, taking the one nearest to the wire crossing

In August 2016, we selected 21 shoots, distributed regularly, from each pot for clone identification. To achieve the regular pattern, a wire comb‐like structure was slid onto the pot at the substrate–surface level, from two sides to form a grid that was used to identify the position of shoots to be harvested, taking the one nearest to the wire crossing (Figure 2). The sampled shoots were labeled, and a small piece of leaf (0.5 cm^2^) or 5‐cm‐long leaf segment was taken from each shoot to be analyzed by means of flow cytometry or molecular analyses, respectively, for clone identification (see below). This sampling preceded the harvest to ensure that the material will be green enough to make these analyses possible.

All plants, the above‐mentioned 21 shoots individually and then rest of the pot together, were harvested between 9 September and 18 October 2016 using the wire–grid as described above. The 21 shoots were cut at 1 cm above the ground, and their height was measured. Shoots were dried to constant weight at 60°C for 24 hr and individually weighed (those that were damaged between leaf sampling and harvest were excluded from shoot measurements because their height and biomass could not be recorded). The remaining shoots in each pot were also cut at 1 cm above the ground and counted; their biomass was weighed fresh due to its enormous amount and limited drying space. The aboveground fresh biomass of each of the two clones in the pot was estimated by dividing the total pot biomass according to the ratio calculated from the total dry weight of each clone based on the 21 stems sampled individually. Only pots in which both competing clones survived until the end of the 2015 growing season were included in the analysis, because initial mortality was caused by poor establishment and not by the competition. In total, 498 out of the established 612 pots were included. At the end of the experiment in 2016, we did not detect (by analyzing the 21 sample stems per pot by means of flow cytometry or molecular analyses; see Section 2.4) one of the competitors in 88 out of 498 pots (17.6%), which indicates that in those pots, one of the competitors was most likely excluded. These pots were included in the analyses. For numbers of replicates for origin, ploidy level, invasive status, and genome size category, see Table 1 and Table S1.

Differences in plant performance were examined using three characteristics, and comparisons were made within the pairs of clones sharing a pot: (a) relative proportion of clone shoots out of their total count analyzed for identity in the pot (*n* = 21), (b) ratio of aboveground biomass (log‐transformed), and (c) the intensity of competition among the clones expressed by using the relative yield total (RYT), an index based on relating the biomass of each clone in competition to the biomass of that clone when grown alone (e.g., Weigelt & Joliffe, 2003). For the latter analysis, we used log (1/RYT) as a measure representing the intensity of competition among the two compared clones. This is because RYT is essentially a measure of overyielding, so the smaller it is, the stronger the competition effect; hence, the inverse value is used.

### 2.4 Clone identity determination

In pots with combinations including clones with contrasting ploidies and/or absolute genome sizes, affiliation of 21 shoots to a particular clone (chosen by using the wire–grid; see Figure 2) was determined by measuring the size of their genome. For absolute genome size (small/large determination in tetraploids), we used the flow cytometry procedure described above for choosing experimental clones. For inferring ploidy level, we used the same procedure only with DAPI as a fluorochrome and the samples were run at the Sysmex/Partec CyFlow ML instrument equipped with UV‐LED (for details, see Doležel, Greilhuber, & Suda, 2007).

Pots with clones of the same ploidy level and undistinguishable by differences in genome size were analyzed using microsatellites, to estimate the spread of the clones within the pots. Genomic DNA from 21 samples of *P. australis* collected in each pot was extracted from silica gel‐dried leaves using the DNeasy 96 Plant Kit (Qiagen) following the manufacturer's protocol. In total, the DNA was extracted from 2,330 samples. The concentration was estimated using NanoDrop 1000 (Thermo Fisher Scientific), and the quality was checked by gel electrophoresis on 1% agarose gel. DNA of all samples was diluted to equal concentration of 10 ng/µl. For microsatellites analysis, 10 primers from Saltonstall (2003) were tested for all mother plants and five variable primers were selected for further analysis (see Table S2). PCR amplification using Qiagen Multiplex PCR Kit (Qiagen) was carried out in 5 µl reaction containing 1 µl DNA (10 ng/µl), 0.5 µl ddH_2_O, 2.5 µl 2 × QIA MasterMix, 0.5 µl QIA Solution, and 0.5 µl 10 × 2 µM mix of all primers, and using the following temperature profile: 95°C for 5 min; 35 cycles of 95°C for 30 s, 60°C for 90 s, and 75°C for 30 s; and 68°C for 10 min. PCR performance was assessed by gel electrophoresis. The amplified PCR products were separated and visualized via automated capillary sequencing instruments at the Laboratory of DNA Sequencing at the Faculty of Science at Charles University (https://www.natur.cuni.cz/biologie/servisni-laboratore/laborator-sekvenace-dna) using 16‐capillary 3130xl Genetic Analyser (Applied Biosystems). Electropherograms from the fragment analysis were analyzed using GeneMarker software version 2.4.0 (SoftGenetics) with manual corrections. Samples were assigned to maternal genotypes prior to the check of the plant combination within each pot. All of the 21 samples per pot were determined for their clone membership, and their relative spread was estimated.

### 2.5 Statistical analysis

#### 2.5.1 Overview of the datasets and models used

Our aim was to test the effect of genome size (expressed as two categories: small and large), ploidy level (tetraploid, hexaploid, or octoploid), status (NA‐native, NA‐invasive, and EU‐native), and their interactions on plant competition measured at pot and shoot levels. For competition performance at the shoot level, we included the biomass of individual shoots, while at the pot level, it was ratio (target/competitor) of clone stems, and ratio of the clones' aboveground biomass and competition intensity (1/RYT). As all combinations of clone status, ploidy, and genome size were not available, we divided the data into two datasets and tested separately the effect of ploidy and that of genome size, and their respective interactions with status. Therefore, first, the effect of the ploidy level (tetraploid, hexaploid, or octoploid) was tested only in clones with EU‐native and NA‐invasive status (higher ploidies in NA‐native do not occur; see Table 1). Second, the effects of genome size, status, and their interaction were tested only in tetraploids.

#### 2.5.2 Data analysis at the pot level

We explored the effect of clone status (EU‐native vs. NA‐invasive) in an interaction with ploidy level (tetraploid, hexaploid, or octoploid) on the competitive performance of two clones grown together in pots. Pots including NA‐native clones were excluded from analyses because they are all tetraploid. As the chosen performance characteristics involved both clones present in each pot, we used as predictors the categorical or numerical variables comparing the clones. For clone status, a categorical variable *statusComp* was defined with levels *InvInv* (when two invasive clones were compared), *NatNat* (when two native European clones were compared), and *InvNat* (when comparing an invasive clone with a native European clone). To characterize the difference in ploidy, we have used a numerical variable *ploidyComp*, representing a log‐transformed ratio of nominal ploidy levels (e.g., log(6/4) when a hexaploid clone is grown with tetraploid one). Note that as most performance characteristics have a direction (e.g., ratios of shoot counts or aboveground biomasses, resulting in negative values when the trait value in focal clone was lower than that for a competitor), even the *ploidyComp* variable is signed.

Further, we investigated the effect of clone status (EU‐native vs. NA‐native vs. NA‐invasive) for tetraploids only, but within tetraploids, we compared clones with large and small genomes. Clone status was represented by a categorical variable *OrStatComp*, representing a combination of origin and status of competing clones, with six levels (*NAinvNAinv*, *NAinvNAnat*, *NAinvEUnat*, *NAnatNAnat*, *NAnatEUnat*, and *EUnatEUnat*). The predictor characterizing genome size of the two clones (*gscatComp*) was again a categorical variable with four levels (*lrglrg*, *lrgsml*, *smllrg*, and *smlsml*).

Depending on the chosen response variable (performance characteristic) type, we used either a generalized linear model (for a ratio of shoot counts, with assumed binomial distribution with explicitly modeled overdispersion) or a general linear model (for the log‐transformed ratio of clone fresh aboveground biomasses and for the log‐transformed inverse value of RYT, quantifying the intensity of competition). We started by fitting a model with main effects of both *statusComp* and *ploidyComp*, subsequently eliminating nonsignificant one(s) based on a statistical test. If at least one of the main effects was retained, we also tested the interaction between *statusComp* and *ploidyComp*, corresponding to a hypothesis that the difference between North American invasive and native European clones varies with ploidy level. When comparing the tetraploid clones in the second set of analyses, we used the same approach as described in the previous paragraph, with a categorical *gscatComp* replacing the numerical *ploidyComp* predictor.

#### 2.5.3 Data analysis at shoot level

To address the difference between tetraploid clones of different status and genome size, we analyzed the biomass of individual shoots sampled from the pots with competing clones. As the shoot observations coming from the same pot are not independent, we used a linear mixed‐effects model (LMM) with the shoot biomass log‐transformed (to achieve homogeneity of variances) and with a random effect of pot specified as affecting the model intercept. Because at the level of shoots each case belongs to a single clone, we conservatively considered the effects of clone properties in our models before examining the additional effects of property combinations of the two competing clones. For clones, we considered two characteristics and their interaction: clone status in *OrStat* (with three levels: *EUnat*, *NAinv*, and *NAnat*) and genome size categorical variable *gscat* (with levels *large* and *small*). After selecting significant terms based on those two characteristics, we examined possible extension of the model with *OrStatComp* and *gscatComp* predictors (and then, when at least one of the predictors was significant, with their interaction), as described in the preceding section. In the context of LMM, the tests of model terms were performed using likelihood‐ratio test (LRT).

#### 2.5.4 Additional methods and software used

All statistical models were estimated in the R software version 3.5.1 (R Core Team, 2018). LMMs were fitted using the *lme4* package (Bates, Maechler, Bolker, & Walker, 2015). To visualize the effects of selected predictors in our models, the *effects* package (Fox & Weisbert, 2018) was used, while the multiple comparisons among the levels of significant categorical variables were performed with the *multcomp* package (Bretz, Hothorn, & Westfall, 2010).

## 3 RESULTS

### 3.1 Effects of genome size on the performance of tetraploid clones of different status

**Figure 3:**
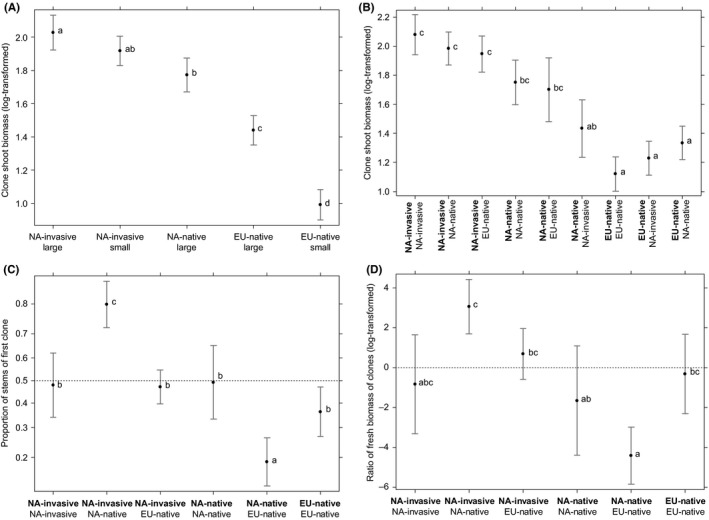
(A) Clone shoot biomass in relation to status (NA‐invasive, NA‐native, EU‐native) and genome size of the competitor. One combination (NA‐native with small genome size) is missing as it does not occur in nature. (B) Target clone shoot biomass (listed first, in bold) in relation to its status and that of its competitor. (C) Proportion of shoots of target and competitor clone in relation to their status. Target clone listed first in bold. (D) Ratio of target and competitor clone fresh biomass in relation to their status. Target clone listed first in bold. Group means are based on estimated models and are shown with 95% confidence intervals. Only tetraploid clones are included in these comparisons

There were significant differences among clones due to their status (EU‐native, NA‐invasive, and NA‐native) and due to their genome size, but there was also a significant interaction between both factors (Table 2). The nature of those effects is shown in Figure 3A. NA‐invasive clones had the largest average shoot biomass, but with only a limited, nonsignificant difference due to genome size. The shoot biomass of NA‐native clones (which have only a large genome) was intermediate between NA‐invasive and EU‐native. The shoots of EU‐native clones were the shortest, and their biomass was smaller in clones with small genomes than with large genomes.

**Table 2:** Summary of model describing the differences in shoot biomass as affected by status and genome size category (large vs. small) of the tetraploid clone to which a shoot belongs (*Status*, *Genome Size*, and their interaction *Status:Genome Size*), as well as by the predictors comparing the two clones co‐occurring in a pot (*StatusComp*, *Genome SizeComp*, and their interaction *StatusComp:Genome SizeComp*)

Predictor	Model	Shoot biomass	
		LMM with log(biomass)	
	*df*	*χ* ^2^	*p*
Status	2	219.6	<.001
Genome size	1	37.9	<.001
Status:Genome size	1	12.9	<.001
StatusComp	6	15.1	<.020
Genome SizeComp	2	0.04	n. s.
StatusComp:Genome SizeComp	14	10.8	n. s.

Note

The *df* column shows the corresponding degrees of freedom for each tested term, the *χ* ^2^ column shows values of the test statistic used in the likelihood‐ratio test, and the *p* column gives a type I error estimate. The model used *n* = 3,422 shoots, collected from 182 pots and 312 unique pot:clone combinations.

Shoot biomass of two competing clones was not affected by differences in their genome size; the only significant effect was that of their status in combination with status of the competing clone (Table 2, bottom part); specifically, the shoot biomass of the NA‐native clones differed in response to the status of the competing clone (Figure 3B).

At the pot level, differences in genome size had no significant effect on the clone performance (Table 3). All the differences in the relative performance that we detected depended on the status of the competing clones. The shoot ratios (Figure 3C) did not differ between two competing NA‐invasive clones, or between two NA‐native, or between two EU‐native clones. Similarly, the clones performed equally when a NA‐invasive clone competed with an EU‐native. However, NA‐native clones had lower shoot counts, both when competing with a NA‐invasive or EU‐native clone (Figure 3C). Analogous patterns can be seen in the ratio of aboveground fresh biomass of the two clones (Figure 3D). Only when a NA‐native clone competed with one of the two other clone types (NA‐invasive or EU‐native), the biomass ratio was significantly different from 1, always at the expense of the NA‐native clone.

**Table 3:** Summary of models describing the effects of clone status and genome size category (large vs. small, only tetraploids compared) on the proportion of shoot counts and ratios of aboveground biomass

Predictor	Model	Proportion of shoot counts		Ratio of aboveground biomass	
		GLM (binomial)		LM with log(ratio)	
	*df*	*F*	*p*	*F*	*p*
StatusComp	5, 139	14.49	<.001	8.01	<.001
Genome SizeComp	3, 139	0.08	n. s.	0.07	n. s.
StatusComp:Genome SizeComp	11, 131	0.27	n. s.	0.60	n. s.

Note

Columns labeled with *F* contain values of the *F* statistic, and those labeled with *p*, the estimated significance of the test. The *df* column shows corresponding degrees of freedom for each tested term. Note that the labels *StatusComp* and *Genome SizeComp* refer here to predictors describing, respectively, the combination of the status and combination of genome size for the two clones grown together. Fitted models used *n* = 152 pots.

### 3.2 Effects of ploidy on the performance of EU‐native and NA‐invasive clones

**Figure 4:**
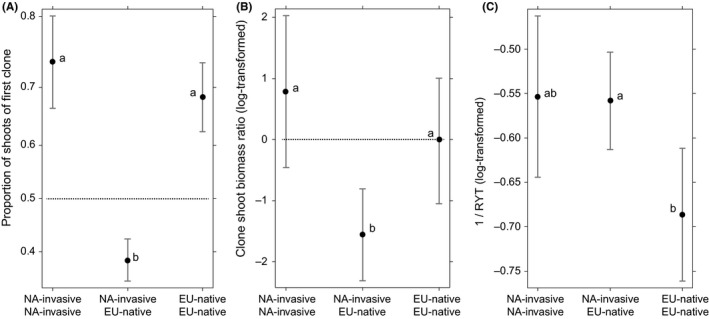
Results of competition expressed as: (A) the proportion of shoots produced by the target clone and the competitor (target clone listed first, in bold). (B) Log‐transformed ratio of clone biomasses. (C) Intensity of aboveground competition, the higher the value, the stronger the competition. Group means are based on estimated models and are shown with 95% confidence intervals. All ploidy levels of EU‐natives and NA‐invasives are included in these comparisons. For (A) and (B), the expected outcome for an equal competitive strength of both clones is indicated by dotted horizontal line

There was no effect of ploidy, not even depending on the clone status, on any of the characteristics examined: shoot ratio of the competing clones, their aboveground biomass ratio, or intensity of competition expressed as an inverse value of RYT (Table 4). The only significant effect was status—NA‐invasive clones competing with EU‐native clones performed worse in terms of both shoot count (Figure 4A) and the total aboveground biomass (Figure 4B). The competition asymmetry in shoot counts was greater for combination of the same status, NA‐native and EU‐native, than for NA‐invasive with EU‐native. The intensity of competition was significantly lower when two EU‐native clones competed with each other, compared to pots with NA‐invasive clone involved (Figure 4C).

**Table 4:** Summary of models describing the effects of clone status and ploidy upon three performance characteristics

Predictor	Model	Proportion of shoot counts		Ratio of aboveground biomasses		Competition intensity (1/RYT)	
		GLM (binomial)		LM with log(ratio)		LM with log(1/RYT)	
	*df*	*F*	*p*	*F*	*p*	*F*	*p*
StatusComp	2, 296	46.27	<.001	6.04	.003	4.15	.017
PloidyComp	1, 296	0.16	n. s.	0.34	n. s.	0.03	n. s.
StatusComp:PloidyComp	3, 294	0.75	n. s.	0.61	n. s.	0.75	n. s.

Note

The *df* column shows corresponding degrees of freedom for each tested term. Columns labeled with *F* contain values of the *F* statistic, and those labeled with *p*, the estimated significance of the test. Note that the labels *StatusComp* and *PloidyComp* refer here to predictors describing, respectively, the combination of the status for the two clones grown together and the difference of their ploidy levels. Fitted models used *n* = 300 pots.

## 4 DISCUSSION

### 4.1 Rationale of the study

This study made use of an experimental system consisting of populations, represented by particular clones and belonging to three phylogeographic groups (lineages) of *Phragmites australis*, an ecologically important wetland dominant—native North American plants, invasive populations from Europe that are apparently replacing them, and native European populations. Such experimental arrangement allowed us to obtain insights into the mechanisms of their coexistence in the field and into possible changes in performance of invasive populations after more than hundred years since their introduction. Besides the primary test of competitive relationships between these three groups, performed in standardized conditions of a temperate common garden, we focused on cytological characteristics of individual populations. We hypothesized that populations with small genomes would be more competitive as this trait was shown to be associated with invasiveness (Pyšek et al., 2018; Suda et al., 2015) in *P. australis* and in another grass, *Phalaris arundinacea* (Lavergne et al., 2010, but see Martinez et al., 2018, who recently questioned the results of that study). Further, high ploidies are known to be competitively superior over low ploidy levels (Pandit et al., 2014; te Beest et al., 2012); hence, we hypothesized that tetraploids will be competitively weaker than hexa‐ and octoploids.

So far, the research in the role of genome size in invasiveness has been mostly based on indirect evidence, by comparing performance of plants with different genome sizes (Lavergne et al., 2010; Meyerson, Cronin, & Pyšek, 2016; Pyšek et al., 2018) and by exploratory analyses among species (Kubešová et al., 2010; Pandit et al., 2014; Suda et al., 2015). Here, we performed a direct test of intraspecific competition on the populations of *P. australis* of different status and invasion history to reveal how these characteristics interact with genome size and ploidy levels to affect competition outcomes. Moreover, this is the first experimental test of competitiveness of individual populations of *P. australis*—and competition may be assumed to play a key role in the invasion of North American wetlands, where native and invasive populations co‐occur in the same habitats (Meyerson et al., 2009; Packer et al., 2017).

### 4.2 Outcome of competition is determined by invasion status of competing clones

Overall, our results illustrate that the performance of populations in competition primarily depended on their status. Measured by shoot number or aboveground biomass, the strongest signal observed across various combinations of clone status was that only if North American native populations were involved in competition, winners and losers could be identified, with the result always at the expense of the North American native populations. The effect of invasion status that we observed is in accordance with studies showing the competitive superiority of invasive species over native (see e.g., Gioria & Osborne, 2014; Goldstein & Suding, 2014, and references therein), the novelty of our research being that we confirmed this mechanism acting at the within‐species population level.

Another important observation, measured by shoot count and aboveground biomass, is that the North American native populations were suppressed by European natives to a similar degree as by North American invasives. The fact that European native populations are such a strong competitor against North American native plants indicates that competition might have been involved in the initial invasion centuries ago, following introduction, and in facilitating the initial space preemption in sites that European plants reached by long‐distance dispersal. Here, it needs to be noted that the European native populations have an even smaller genome than North American invasive, as shown in the previous common‐garden experiment (Pyšek et al., 2019). Pyšek et al. (2018) hypothesized about a possible postintroduction shift in traits, providing European invaders with advantages at different stages of the invasion process, and interpreted these findings with respect to introductions of populations with different genome sizes. According to these authors, among the European populations introduced to North America, those that established and spread likely had on average slightly bigger genomes than those that might have been filtered out following introduction from the native European range. In the initial stage of invasion, bigger genomes might have proven advantageous as they are associated with traits favoring spread, such as increased allocation to generative reproduction (Pyšek et al., 2018). Yet, relative to the native North American *P. australis* populations, the genomes of the European populations that became invasive in North America were comparatively small enough to generate trait differences that provided the invading populations with competitive superiority over the native populations (Pyšek et al., 2018). Another reason for the success of invasive populations could be that the smaller genome size allowed them to thrive in a wider range of conditions (Suda et al., 2015). The niche of native and invasive *P. australis* populations in North America is not an exact overlap, the invasive niche is much greater for a variety of abiotic conditions ultimately allowing greater propagule pressure (Meyerson et al., 2009).

### 4.3 No effect of genome size and ploidy level on competition

Overall, the effects of genome size on the outcome of competition in our experiment were difficult to detect and manifested mainly through the shoot biomass of European native populations—this illustrates that the clone status is the most important predictor of the outcome of competition, especially given the variation in measured characteristics and logistically limited numbers of replicates used in the experiment. That the effect of genome size was only detectable for European native populations, rather than for both groups occurring in the North America, might be related to the extent of variation within source populations. The variation in genome size of North American native populations is rather restricted (Pyšek et al., 2018), which may explain why this trait had little effect, if any, on growth of clones representing this group. In contrast, the variation in European native populations is broader, ranging from small to large genomes, thereby creating more opportunity for the relationship between genome size and shoot biomass to manifest and be detected. However, it needs to be kept in mind that these weak effects were only demonstrated at the level of individual shoots, providing thus a limited indication of the population's competitive strength—at the whole‐pot level, the interaction of genome size with invasion status was not significant.

The competitive relationships among ploidy levels other than tetraploids (that were used in the experiment aimed at testing the effect of genome size) did not reveal any effect of ploidy level on the outcome of competition.

### 4.4 Competition within North American invasives is strongest, but they lose against their European ancestors

In terms of clone status, the asymmetry of competition was most pronounced in pairs with two North American invasive populations competing, but when grown in competition with European natives, North American invasive populations were inferior. However, it should be noted that this difference was significant only in models with all ploidy levels considered (comparing EU‐native vs. NA‐invasive), but the significance disappeared in comparisons of tetraploids only. Therefore, we cannot exclude the possibility that this discrepancy is due to greater power with the increased number of samples. Nevertheless, this is a potentially interesting result because North American invasive populations grew taller and produced greater biomass than most other groups, including European natives (with the exception of European native octoploids, which are not inferior in terms of productivity; te Beest et al., 2012). Increased vigor in octoploids is reported by Hansen, Lambertini, Jampeetong, and Brix (2007) and Achenbach et al. (2012) who compared populations with different ploidy levels in two distinct common‐garden experiments. However, they found vigorous octoploids only in the Danube Delta in Europe, while other European or Asian populations performed worse than tetraploids (Achenbach et al., 2012; Hansen et al., 2007). This supports our findings of rather unclear effect of ploidy, which strongly depends on the number and characteristics of the compared populations.

North American invasives are also the most aggressive when competing with each other but lose in direct competition with their European native ancestors. One possible explanation of this result could be that the variation in the competitive strength of North American invasive populations might be greater, reflecting the invasion into less competitive stands of generally weaker North American native populations. This would allow establishment and spread of populations that were less competitive, but with good abilities for seed dispersal in the initial colonizing stage of invasions (Pyšek et al., 2018). Such populations may not be successful in highly productive wetlands in Europe dominated by *P. australis* (Ellenberg, 1988) where only competitive populations would survive. This is in accordance with the results of Guo, Lambertini, Nguyen, Li, and Brix (2014), Guo, Lambertini, Pyšek, Meyerson, and Brix (2018) who showed that the populations invading in North America may have arrived on this continent preadapted from Europe and experience further postintroduction evolution in response to the new environment.

### 4.5 Inferior competitive ability of native wetland dominant: seeds of future threat

It needs to be kept in mind that the results we report in this paper are based on a common‐garden experiment conducted in a single temperate garden, which somewhat limits generalization of our findings, as with many ecological experiments. Nevertheless, the clones used in competition represent populations originating from reasonably similar climatic regions in both Europe and North America, and the effect of local soil was filtered out by using sand as a neutral substrate. We thus did not aim to address the effects of soil properties or varying levels of moisture on the outcome of competition, that is, factors that would possibly influence the results. Yet, our paper is the first providing fairly robust evidence that North American native populations are likely to lose in direct competition with European plants, be it those already introduced to North America or potentially introduced in the future. In this respect, it is important to realize that European native populations, whose effect on North American native populations is even stronger than of those co‐occurring with North American natives for centuries now, are still potential sources of future vigorous invaders of North American wetlands. As we show, these new introductions are likely to succeed in competition with native common reed populations. This represents an ongoing threat to wetland biodiversity on this continent.

## CONFLICT OF INTEREST

None declared.

## AUTHORS' CONTRIBUTIONS

PP, JČ, HS, and LAM conceived and designed the research. ZC, CL, ML, HR, PT, and KŠ collected and prepared the data. PŠ and JČ analyzed the data. PP, JČ, and PŠ wrote the paper. All authors commented on the manuscript.

## Data Availability

Data are available from Dryad Digital Repository: https://https//doi.org/10.5061/dryad.stqjq2c00.
