# Supplementary material for: Competition among native and invasive Phragmites australis populations: An experimental test of the effects of invasion status, genome size, and ploidy level
Source: Ecol Evol. 2020 Jan 13;10(3):1106–18. doi: 10.1002/ece3.5907 (PMC7029062; doi:10.1002/ece3.5907)
Supplement: Supplementary file 1 [file ECE3-10-1106-s001.docx]

Figure S1. Location of the clones used in the study (see Table 1 for details).


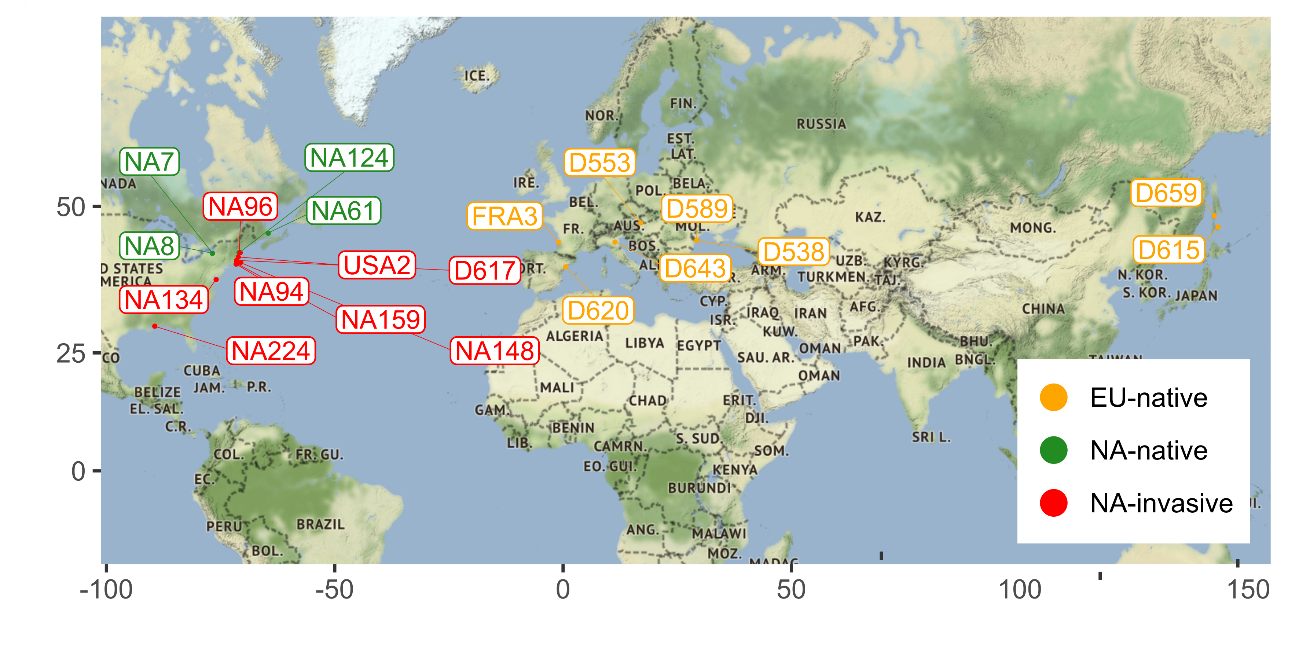


Table S1. Number of replicates for competitor pairs. Numbers show combination of clones of different origin (EU-Europe, NA-North America), status (N-native, I-invasive), ploidy level (8,6,4x), and genome size category of 4x (L-large, S-small) at the harvest (only pots with both competitors alive were included).

|  | EU-N-8 | NA-I-8 | EU-N-6 | NA-I-6 | EU-N-4L | EU-N-4S | NA-I-4L | NA-I-4S | NA-N-4L |
| --- | --- | --- | --- | --- | --- | --- | --- | --- | --- |
| EU-N-8 | 9 | 11 | 12 | 8 | 12 | 12 | 9 | 12 | 19 |
| NA-I-8 |  | 9 | 10 | 8 | 11 | 12 | 9 | 12 | 19 |
| EU-N-6 |  |  | 9 | 11 | 12 | 12 | 5 | 12 | 19 |
| NA-I-6 |  |  |  | 5 | 8 | 7 | 6 | 6 | 10 |
| EU-N-4L |  |  |  |  | 8 | 12 | 11 | 12 | 17 |
| EU-N-4S |  |  |  |  |  | 9 | 8 | 12 | 16 |
| NA-I-4L |  |  |  |  |  |  | 6 | 7 | 16 |
| NA-I-4S |  |  |  |  |  |  |  | 9 | 19 |
| NA-N-4L |  |  |  |  |  |  |  |  | 19 |

Table S2. Five variable primers selected for microsatellite analysis (according to Saltonstall 2003).

| fluorescent dye | 6FAM | NED | VIC | PET | VIC |
| --- | --- | --- | --- | --- | --- |
| locus | PaGT 8 | PaGT 9 | PaGT 12 | PaGT 14 | PaGT 16 |
| repeat units | (CA)9 | (CA)10 | (CA)9 | (CA)7 | (CA)10 |
| forward primer | TCTGAACATAATCCTGGTGG | CCATGTGTTAATGTTGTCC | CTTCCTAGGTCAGTATCATCC | GTTGCAGCAAGTATTTGG | ACCAATCAGTCAGACTAGCC |
| reverse primer | TCTGTGTGAAGCAGTTCTGC | ATTGAATCCACACGTTTCCG | GTGGCAGCTGATTGATTTGG | CAAGCATTCTAGTAGTAGC | GTTCTCATGTTGGAGAAGCC |
| size range | 170-193 | 188-224 | 151-196 | 169-198 | 231-298 |

Saltonstall, K. (2003) Microsatellite variation within and among North American lineages of *Phragmites* *australis*. *Molecular Ecology*, 12, 1689-1702.
